# Standards of care and determinants of women’s satisfaction with delivery services in Nepal: a multi-perspective analysis using data from a health facility-based survey

**DOI:** 10.1186/s12884-024-06301-9

**Published:** 2024-02-13

**Authors:** Sabita Tuladhar, Maria Delius, Matthias Siebeck, Cornelia Oberhauser, Deepak Paudel, Eva Rehfuess

**Affiliations:** 1 Teaching & Training Unit, Division of Infectious Diseases and Tropical Medicine, University Hospital, LMU, Munich, Germany; 2 Center for International Health, LMU, Munich, Germany; 3 Department of Obstetrics and Gynecology, University Hospital, LMU, Munich, Germany; 4 Institute of Medical Education, LMU University Hospital, LMU, Munich, Germany; 5 Institute for Medical Information Processing, Biometry, and Epidemiology, LMU, Munich, Germany; 6 Pettenkofer School of Public Health, Munich, Germany; 7 Save the Children, Kathmandu, Nepal

**Keywords:** Maternal health, Maternal mortality, Standards of care, Quality of care, Health facility survey, Delivery care, Quality, Low- and middle-income country

## Abstract

**Background:**

Compliance with standards of care is required for sustained improvement in the quality of delivery services. It thus represents a key challenge to improving maternal survival and meeting the Sustainable Development Goal (SDG) target of reducing the maternal mortality ratio to 70 deaths per 100,000 live births. This study examines the extent to which normal low-risk health facility deliveries in Nepal meet the standards of quality of care and assesses the effect of the standards of quality of care and various contextual factors on women’s satisfaction with the services they receive.

**Methods:**

Drawing on the 2021 Nepal Health Facility Survey, the sample comprised 320 women who used health facilities for normal, low-risk delivery services. A weighted one-sample t-test was applied to examine the proportion of deliveries meeting the eight standards of care. Women’s overall satisfaction level was computed from seven satisfaction variables measured on a Likert scale, using principal component analysis. The composite measure was then dichotomized. Binary logistic regression was used to analyze the determinants of women’s satisfaction with delivery care services.

**Results:**

Deliveries complying with the eight standards of care and its 53 indicators varied widely; output indicators were more frequently met than input indicators. Of the eight standards of care, the “functional referral system” performed highest (92.0%), while “competent, motivated human resources” performed the least (52.4%). Women who were attended by a provider when they called for support (AOR: 5.29; CI: 1.18, 23.64), who delivered in health facilities that displayed health statistics (AOR 3.16; CI: 1.87, 5.33), who experienced caring behaviors from providers (AOR: 2.59; CI: 1.06, 6.30) and who enjoyed audio-visual privacy (AOR 2.13; CI: 1.04, 4.38) had higher satisfaction levels compared to their counterparts. The implementation of the Maternity Incentive Scheme and presence of a maternal waiting room in health facilities, however, were associated with lower satisfaction levels.

**Conclusions:**

Nepal performed moderately well in meeting the standards of care for normal, low-risk deliveries. To meet the SDG target Nepal must accelerate progress. It needs to focus on people-centered quality improvement to routinely assess the standards of care, mobilize available resources, improve coordination among the three tiers of government, and implement high-impact programs.

**Supplementary Information:**

The online version contains supplementary material available at 10.1186/s12884-024-06301-9.

## Background

Maternal survival has been a global priority for more than three decades, as illustrated by United Nations (UN) Millennium Development Goal (MDG) 5, which aimed to reduce maternal mortality by three-quarters between 1990 and 2015, and Sustainable Development Goal (SDG) 3.1, which seeks to reduce the maternal mortality ratio (MMR) to 70 deaths per 100,000 live births by 2030 [1]. Despite efforts to improve access to skilled birth attendance and health facility (HF)-based interventions, most low- and middle-income countries (LMICs) did not meet MDG 5 [2] and are challenged to achieve the SDG 3.1 target.

Nepal was close to meeting MDG 5 [3], and over the past two and a half decades the country has made significant progress in improving access to HF-based delivery services, as demonstrated by an increase in HF births from 8.0 to 79.3% between 1996 and 2022 [4]. However, an increase in access to HF-based delivery services does not always translate to high-quality obstetric care and better maternal survival. With nearly 650,000 Nepalese women getting pregnant annually [5], about half of the total HFs offering normal low-risk delivery services in 2021 [6], and 79.3% of pregnant women giving birth at a HF in 2022 [4], Nepal still faces the challenge of a high MMR of 151 deaths per 100,000 live births in 2021, with 57.0% of these deaths occurring at HFs [7]. Of the total deceased women, 56.0, 38.0, and 3.0% had normal low-risk, caesarean, and assisted deliveries, respectively, while the mode of delivery of 3.0% of the women was unknown [7]. To meet the SDG 2030 3.1 target, Nepal needs to reduce the MMR by around 9.0% every year, which requires significant improvements in the quality of HF-based delivery services along with implementing evidence-based and effective maternal health interventions, in addition to ensuring that all women in Nepal have access to such services.

Quality of care (QoC) is multi-dimensional, contextual, and subjective, which makes it challenging to define and measure this construct. The QoC framework developed by the World Health Organization (WHO) builds on previous QoC models and identifies eight domains of QoC: i) evidence-based practices for routine care and management of complications; ii) actionable information systems; iii) functional referral systems; iv) effective communication; v) respect and preservation of dignity; vi) emotional support; vii) competent, motivated human resources; and viii) essential physical resources; these domains need to be regularly monitored and improved [8]. The framework recognizes the differences between the provision of care and the experience of care, as well as the interlinkages between the two, and takes account of the structure, processes, and outcomes of care. Corresponding to the eight domains of the QoC framework, WHO formulated eight standards of care which are further detailed as quality statements and quality measures [9]. These standards of care explicitly define what is required to achieve high-quality care around the time of childbirth. They also account for the critical role of communities and clients and their needs and preferences with regards to managing their own health, in addition to the care provided in distinct HFs [9].

Nepal’s political transition in 2017 from a unitary government system to a federalized system with three tiers of government – federal, provincial, and local – presents a novel opportunity for the 753 local governments to re-focus on the provision of high-quality basic healthcare services free of cost, as mandated by the constitution. However, several challenges exist when trying to meet this constitutional mandate, largely due to unclear roles and responsibilities of the three tiers of government, among them poor budget allocation processes and limited human resource capacity at local levels directly affect the provision of health services and their quality [10]. Moreover, the coronavirus disease-19 (COVID-19) pandemic that hit Nepal in 2020 put the health system under significant pressure. Several health indicators were impacted negatively, including those relating to maternal health [11].

To fulfill the MDG 5 commitment, Nepal prioritized maternal health and developed and implemented several strategies and programs for providing safe motherhood services to women. The Maternity Incentive Scheme (*Aama Surakshya Karyakram*) implemented across the country since 2009 has been foundational in addressing critical barriers to HF-based births where pregnant women receive delivery services free of cost, are entitled to receive a transportation allowance for completing antenatal visits at HFs in the fourth, sixth, eighth, and ninth months of pregnancy, and for giving birth at a HF, while the HFs are reimbursed for the cost of every delivery. The transportation allowance for women varies between plain, hill and mountain regions of the country while the reimbursement for a delivery varies by the type of delivery: normal-low risk deliveries, deliveries with complications, and cesarean deliveries [12]. The gradual scale-up of the Maternal and Perinatal Deaths Surveillance and Response System reached 95 out of a total of 561 hospitals in 2020 (i.e., 16.9%), and has been key in identifying gaps in QoC and implementing actions to address these gaps [5]. However, efforts to date have not resulted in adequate and sustained improvements in QoC. The results of the national-level HF-based surveys, the routine implementation of the HF-based Minimum Service Standards (MSS) readiness assessment tools, and other studies have consistently shown low compliance with standard processes, as well as low job motivation among service providers [6]. A recent HF-based study comparing data of 2015 and 2021 identified slow progress in HF readiness, including a lack of delivery care guidelines and basic equipment, high stock-outs of essential medicines, as well as low training among the delivery attendants in both public and private HFs [13]. Improving QoC has been a strategic focus of previous health strategic plans, as well as the recently launched Nepal Health Sector-Strategic Plan (NHS-SP) for the years 2023–2030. The importance of QoC has also been reflected in several national strategies and action plans, notably the Nepal Safe Motherhood and Newborn Health Road Map 2030. The National Medical Standard (NMS) for Maternal and Newborn Care Volume III in 2007, revised in 2009 and 2020, defines operating procedures for maternal and newborn service delivery in Nepal and is aligned with WHO recommended standards of care [14].

In view of the strategic focus on reducing maternal mortality and meeting the standards of care of normal-low risk delivery services for improving the overall quality of delivery services in Nepal, this study has the following two objectives. Firstly, it examines the extent to which normal low-risk HF deliveries in Nepal meet the standards of QoC. Secondly, it assesses the effect of the standards of QoC and various contextual factors on women’s satisfaction with the services they receive.

## Methods

### Study design and population

This cross-sectional study used publicly available data from the 2021 Nepal Health Facility Survey (NHFS) retrieved from https://dhsprogram.com/Data/. It utilized four types of data from the multi-component NHFS: (i) direct observation of labor and delivery services provided to women; (ii) post-partum exit interviews with women as they were discharged from the HF; (iii) HF inventory assessments; and (iv) interviews with the service providers attending labour and delivery. The sample size was determined by the women for whom both components (i) and (ii) could be undertaken; information from components (iii) and (iv) was then linked to these data to complement the analysis. The study thus analyzed data from 320 women whose labor and delivery procedures were observed at HFs and who were then interviewed post-delivery as they were discharged from the HF regarding their experience of care and satisfaction with the services they received.

### Sampling

The 2021 NHFS assessed 1576 HFs in Nepal, of which 804 reported providing normal low-risk delivery services. Normal low-risk deliveries are those that are spontaneous in onset, low risk at the beginning of labor, and remain that way throughout labor and delivery; the infant is born naturally out of the vertex position between 37 and 42 weeks of pregnancy; and after birth, both the mother and the infant are in good health [15]. Details of the 2021 NHFS sampling strategy are reported elsewhere [6]. With regards to this study, the following information is relevant:

#### Observation of women during labour and delivery

The data collectors spent 2 days at each HF to observe women during labour and delivery in all HFs where normal low-risk delivery services were available. Observations could be made in 125 HFs where the data collectors attempted to observe as many women as possible. A total of 457 women were observed during labour and delivery.

#### Exit interviews with post-partum women

The exit interviews were undertaken as the women were discharged from the HF, with the aim to interview all women who were observed for labour and delivery. However, out of the 457 women observed, only 320 in 94 HFs were interviewed. The remaining 137 women could not be interviewed for various reasons, such as that they suffered complications, were referred to another HF, refused to be interviewed, or remained in the same HF for a prolonged period of time.

#### HF inventory assessment

Inventory assessments for normal low-risk delivery services were conducted for all 804 HFs that reported providing normal low-risk delivery services.

#### Interviews with the service providers attending labour and delivery

Interviews with 2,742 providers were undertaken for all 804 HFs that reported providing normal low-risk delivery services.

### Data collection

A validated comprehensive checklist was used for every woman observed during labor and delivery to capture whether the service providers correctly performed key evidence-based interventions during each stage, including the initial client assessment, the first stage of labor, the second and third stages of labor, and the immediate care of the mother and newborn after birth. The exit interview employed a pre-tested questionnaire consisting of questions related to accessing care and decision-making, knowledge of the Maternity Incentive Scheme, perceptions of care, and satisfaction with delivery services. The inventory encompassed a complete review of HF infrastructure, the availability of necessary equipment and medicines for routine deliveries, services offered, basic amenities, infection prevention, and waste disposal practices. The provider interview included pre-tested questions on training, duration of work, work environment, and motivation.

Four weeks of training were provided to nine medical doctors and one nurse with a master’s degree in nursing, who supervised a pool of 135 data collectors who had bachelor of science in nursing, bachelor of nursing, or bachelor in public health degrees. The data collectors received 4 weeks of training. Data on labor and delivery services were collected by the nurses, who were all females. HF inventory, service provider interviews, and exit interviews were conducted by the trained nurses and the public health graduates. Data was collected between January and September 2021 with breaks in May through July due to the COVID-19 imposed lockdown.

### Conceptual framework

**Fig. 1:**
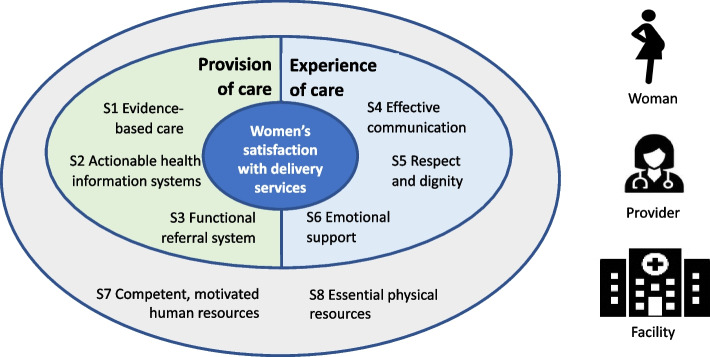
Conceptual framework linking the eight WHO standards for improving maternal care with women’s satisfaction with normal low-risk delivery services in health facilities

Figure 1 presents the conceptual framework of this study, adapted from the WHO Framework for the quality of maternal and newborn health care [8]. Each of the eight standards of care corresponds to the respective domains of the WHO QoC framework. These standards of care capture the provision of care, the experience of care, and the immediate physical environment in which the care is offered; they assess each of the standards of care at the input, output and outcome levels of the results chain using a defined set of indicators. This conceptual framework visualizes the standards of care as two concentric ovals. At the center of the framework is the outcome of interest, i.e., women’s satisfaction with normal low-risk delivery services, which is influenced by the two ovals. The first oval comprises both the provision of care and the experience of care, covering six of the eight standards of care, while the second oval represents the immediate external environment, covering the remaining two standards of care. Three types of contextual factors influencing the outcome of interest – characteristics of women, characteristics of service providers, and characteristics of the HF – are presented in the outer box.

### Unit of analysis

Women, whose labour and delivery were observed and who were then interviewed as they were discharged from the HF, represented the unit of analysis. As described above, data from the HF inventory, the exit interviews and the provider interviews were then linked to these data.

## Study variables

### Outcome variable

This study used women’s satisfaction with normal low-risk delivery services as the outcome variable. During the exit interviews women were asked about seven aspects of QoC they experienced during their stay on the labour and delivery ward. These aspects were measured on a Likert scale from 1 to 5, where 1 represents the highest satisfaction and 5 represents the lowest satisfaction. These aspects referred to i) waiting time, ii) information received from the service provider, iii) skill of the service provider, iv) politeness and empathy of HF staff, v) cleanliness of the HF, vi) privacy, and vii) care received at the HF. The responses to these seven aspects were aggregated into a composite measure of satisfaction using Principal Component Analysis (PCA). Variable loadings on the first principal component that resulted from the PCA were used to compute a composite measure of women’s satisfaction. The variable was then made dichotomous, using the median level to distinguish between higher satisfaction and lower satisfaction.

#### Standards of care variables

As shown in Table 1, a total of 53 variables were used to measure standards of QoC, distinguishing between input (29 indicators) and output (24 indicators) and using the relevant indicators from the 2021 NHFS facility inventory, service provider interviews, observations of labor and delivery, and client exit interviews. The “input indicators” measure the service readiness elements of the standards of care, and the “output indicators” measure the process of labour and delivery care, or the experience of women after any clinical procedure was performed. The number of indicators analyzed within each of the eight standards of care ranged from two for standard S3 “functional referral systems” to 15 for standard S1 “evidence-based practices”; there was no output indicator for standard S3 “functional referral systems”. Although WHO suggests using the same indicator to measure multiple standards of care, this study used each indicator once, only for the most relevant standard of care, to avoid giving undue weight to selected indicators. All indicators were binary variables.

**Table 1:** Standards of care variables

Standards	Results	Indicators measured as availability of equipment or performance of a service
**S1 Evidence-based practices (15)**	**Input (7)**	(i) Health facility with at least one functioning unit of each of seven basic equipment and supplies for mothers: delivery pack, partograph, examination light, blood pressure apparatus, latex gloves, sterilization equipment, delivery bed
		(ii) Health facility with any delivery care guidelines
		(iii) Health facility with at least one provider trained on delivery care
		(iv) Health facility with five essential medicines for mothers: magnesium sulfate, uterotonics, antibiotics, intravenous solution, skin antiseptics
		(v) Health facility with two types of equipment for an assisted delivery: vacuum aspirator, manual vacuum extractor
		(vi) Health facility with three basic types of equipment for newborns: suction apparatus, bag and mask, infant weighing scale
		(vii) Health facility with five essential medicines for newborns: gentamycin, chlorhexidine, tetracycline, amoxicillin, ceftriaxone powder
	**Output (8)**	(i) Provider monitored the mother’s vital signs
		(ii) Provider administered immediate postpartum uterotonic
		(iii) Provider dried, covered, and cleaned the newborn
		(iv) Provider delivered the newborn to the mother’s abdomen
		(v) Provider supported the initiation of early breastfeeding
		(vi) Provider checked newborn breathing and crying
		(vii) Provider helped mother to initiate skin-to-skin contact
		(viii) Provider counseled on postpartum family planning
**S2 Actionable health information systems (5)**	**Input (4)**	(i) Health facility with Maternal and Newborn Health Service Register
		(ii) Health facility with Health Management Information System monthly reports
		(iii) Health facility that displayed health statistics
		(iv) Health facility with Quality Assurance Action Plans
	**Output (1)**	(i) Women who had a completed discharge slip
**S3 Functional referral systems (2)**	**Input (2)**	(i) Health facility with at least one unit of functioning ambulance or emergency transport
		(ii) Health facility with at least one type of communication equipment
	**Output (0)**	None
**S4 Effective communication (6)**	**Input (3)**	(i) Health facility with at least one unit of information materials on maternal care
		(ii) Health facility that received at least one external supervision from federal, provincial, or local authorities in the last 12-month-period before data collection
		(iii) Health facility which had a 24-hour on-call service
	**Output (3)**	(i) Women received postnatal counseling before discharge
		(ii) Provider explained to women about the delivery procedures
		(iii) Provider completed a partograph
**S5 Respect and preservation of dignity (8)**	**Input (2)**	(i) Health facility with physical environment that allows privacy
		(ii) Health facility with a system for collecting clients’ opinions
	**Output (6)**	(i) Woman did not experience use of physical force or abrasive behavior from the provider
		(ii) Woman experienced caring and appropriate behavior from the provider
		(iii) Woman felt comfortable with visual and auditory privacy
		(iv) Woman did not experience discriminatory behavior from the provider
		(v) Woman was attended to by a provider when she called
		(vi) Woman was not scolded by any provider
**S6 Emotional support (3)**	**Input (1)**	(i) Health facility with a maternity waiting room
	**Output (2)**	(i) Woman who was allowed a companion to join her when requested
		(ii) Provider who provided emotional support and reassurance to the woman
**S7 Competent, motivated human resources (5)**	**Input (4)**	(i) Provider received supervision in the last 12 months
		(ii) Health facility that implemented quality assurance activities routinely
		(iii) Provider reported having a written job description
		(iv) Provider reported opportunities for staff promotion
	**Output (1)**	(i) Provider who was experienced (worked more than 1 year)
**S8 Essential physical resources (9)**	**Input (6)**	(i) Health facility with regular source of electricity
		(ii) Health facility with basic water supply in maternity care areas
		(iii) Health facility with one functioning unit of the six-infection prevention and control equipment (surgical masks, waste receptacle, disinfectant, sterilization equipment, aprons/gowns, latex gloves)
		(iv) Health facility with health-care waste management system
		(v) Health facility with a toilet for female clients
		(vi) Health facility with a newborn corner
	**Output (3)**	(i) Women who reported access to drinking water
		(ii) Women who reported access to a toilet
		(iii) Women who reported getting a maternity bed on time
**Total (53)**	**Input (29); Output (24)**	

The number in parenthesis indicates the number of indicators. These indicators were selected from the 2021 Nepal Health Facility Survey data set. The selection of indicators was guided by WHO’s “Standards for Improving the Quality of Maternal and Newborn Care in Health Facilities 2016”

#### Contextual variables

Table 2 presents the 12 contextual variables related to the characteristics of women, service providers, and HFs analyzed in this study. The selection of the indicators was informed by a literature review, as well as the variables available in the 2021 NHFS data set. Of the 12 variables analyzed, six were categorical and six were binary.

**Table 2:** Contextual variables

Characteristics	Indicators
**Women (6)**	i) Age: less than 20 years, 20–34 years, and 35 or more years
	ii) Caste: advantaged which includes Brahmin/Chhetri, and disadvantaged which includes Terai castes, Dalits, Janajatis, and others.
	iii) Education: ever went to school, never went to school
	iv) The number of pregnancies: one, two, three or more
	v) Experience of complications (during current pregnancy): yes, no
	vi) Experience of stillbirths (during previous pregnancies): yes, no
**Provider (2)**	i) Type of provider: doctor, nurse, auxiliary nurse midwife
	ii) Sex of the provider assisting birth: female, male
**Health facility (4)**	i) Type of health facility: public hospital, other public health facility, private hospital
	ii) Health facility that implemented the Maternity Incentive Scheme: yes, no
	iii) Type of site: emergency obstetric and neonatal care (EmONC), basic emergency obstetric and neonatal care (BEmONC), comprehensive emergency obstetric and neonatal care (CEmONC), other
	iv) Distance to health facility: proximal (< 30 minutes’ walking distance from women’s place of residence), semi-proximal (30–60 minutes’ walking distance from women’s place of residence), distal (> 60 minutes’ walking distance)
**Total (12)**	

The number in parenthesis indicates the number of indicators

## Statistical analysis

### Sample characteristics

For contextual variables, weighted frequencies and proportions were presented to show the characteristics of women, providers and HFs.

### Standards of care for normal low-risk health facility deliveries (objective 1)

To examine the proportion of normal low-risk deliveries that meet the standards of care, a weighted one-sample t-test (with a *p*-value = 0.05) was applied. The weighted means of the deliveries that meet each of the individual input and output indicators of the standards of care were calculated, together with their weighted 95% confidence interval (CI). A weighted average score of each of the standards of care was also calculated, which ranged from 0 to 100%.

### Standards of care, contextual factors, and women’s satisfaction with delivery services (objective 2)

For the seven original satisfaction variables as well as the composite, dichotomized variable on women’s satisfaction with normal low-risk delivery services, weighted proportions were used to describe the satisfaction of women with the delivery services received. To examine the effect of the standards of care and contextual factors on women’s satisfaction with delivery care services, this study applied weighted binomial logistic regression. Initially, the multi-collinearity tests of tolerance and variation inflation factor (VIF) tests were carried out to identify and exclude the highly correlated covariates. In the next step, bivariate logistic regression was carried out to examine the independent effect of each of the covariates on the outcome variable, and odds ratios (OR) were calculated. Finally, the multivariate regression model examined the overall influence of the covariates that were significant in the bivariate regression on the outcome variable, and the adjusted odds ratios (AOR) were calculated. A 95% CI and a *p*-value of 0.05 were assumed.

All analyses were weighted to account for the complex, clustered sample design of the 2021 NHFS. The data analysis was conducted using SPSS (IBM SPSS Statistics 25).

## Ethical approval

The 2021 NHFS received ethical approval from the Nepal Health Research Council (NHRC) and obtained written consent from the HF authority, while oral consent was obtained from all participating service providers, and clients or their next of kin before their participation in the survey. NHRC and the Ludwig-Maximilians-Universität (LMU) Ethics Commission, Munich, Germany approved the current study in June 2021.

## Results

### Characteristics of women, service providers, and health facilities

The women’s age ranged from 18 to 36 years, with 7.8% being younger than 20 years (Table 3). Almost one quarter (24.4%) of women belonged to the advantaged caste group, 84.2% had attended school, 44.1 and 31.5% of women were experiencing their first and second pregnancy, respectively, while 24.4% were higher multiparous. 14.3% had experienced complications during their current pregnancy, and 7.1% of women had previously experienced a stillbirth.

**Table 3:** Characteristics of the women included in the study (weighted)

Characteristics of women	Number	Percent
**Age**		
Less than 20 years	25	7.8
20–34 years	282	88.1
35 years and above	13	4.1
**Caste**		
Advantaged	78	24.4
Disadvantaged^a^	242	75.6
**Education**		
Ever went to school	269	84.2
Never went to school	51	15.8
**Number of pregnancies**^**b**^		
One	141	44.1
Two	101	31.5
Three or more	77	24.4
**Experience of complications**	46	14.3
**Experience of stillbirths**	23	7.1
**Total women**	**320**	**100.0**

^**a**^Terai castes: 29.6%; Janajati: 27.3%, Dalits: 11.8%; Others: 6.9%

^**b**^One missing case

As shown in Table 4, more than half of the births (56.2%) were assisted by a nurse, 36.9% by an auxiliary nurse midwife, and 6.9% by a medical doctor. Almost all birth attendants (96.9%) were female. Seven out of 10 women gave birth in a public hospital; a large majority of 85.6% of women delivered at comprehensive emergency obstetric and neonatal service sites; and nearly two-thirds (64.4%) of the women lived within 30 minutes’ walking distance from a HF.

**Table 4:** Characteristics of service providers and health facilities (weighted)

Characteristics of service providers and health facilities	Number	Percent
**Type of provider**		
Doctor	22	6.9
Nurse	180	56.2
Auxiliary Nurse Midwife	118	36.9
**Sex of the provider assisting the birth**		
Female	310	96.9
Male	10	3.1
**Type of health facility**		
Public hospital	228	71.3
Other public health facility	24	7.3
Private hospital	68	21.4
**Implementation of Maternity Incentive Scheme**	269	83.9
**Type of site**		
Emergency obstetric and neonatal care (EmONC)	25	7.7
Basic emergency obstetric and neonatal care (BEmONC)	8	2.4
Comprehensive emergency obstetric and neonatal care (CEmONC)	274	85.6
Other	14	4.3
**Distance to health facility**		
Proximal, within 30 minutes’ walking distance	206	64.4
Semi-proximal, 30–60 minutes’ walking distance	114	35.6
**Total women**	**320**	**100.0**

In the “Type of provider” variable, one Health Assistant has been merged with the Auxiliary Nurse Midwife category

### Standards of care of normal low-risk health facility deliveries (objective 1)

The weighted percentage of deliveries meeting each of the 53 input and output indicators of the standards of care are reported in Additional file 1: Table S1 (with its 95% CI) and Additional file 2: Fig. S1. Standard S1 “evidence-based practice”, which measures the structural components of services around childbirth, is the most comprehensive of the eight standards. Findings show that most deliveries meet essential equipment for mothers and newborns and essential medicines for mothers, and subsequently most mothers received uterotonics, and most newborns received essential newborn care after birth. However, delivery care guidelines and newborn medicines were lacking. Display of health statistics in HFs and presence of quality assurance action plans, the two indicators of S2 “actionable health information systems” was not met for most deliveries. The availability of transportation and communication equipment, a component of S3 “functional referral system” was near-universal. S4 “effective communication” performed moderately well for individual indicators; S5 “respect and preservation of dignity” performed well, for example, 95.7% of women did not experience any discriminatory behavior from the provider, and 93.9% of women were attended to by a provider when called. S6 “emotional support” also performed well: providers allowed most women to have their companion present when requested and offered emotional support and reassurance to them. For most deliveries, compliance with S7 “human resources”, and S8 “physical environment” was poor. Providers did not have a clear job description, and infection prevention and control (IPC) supplies were missing for many deliveries.

**Fig. 2:**
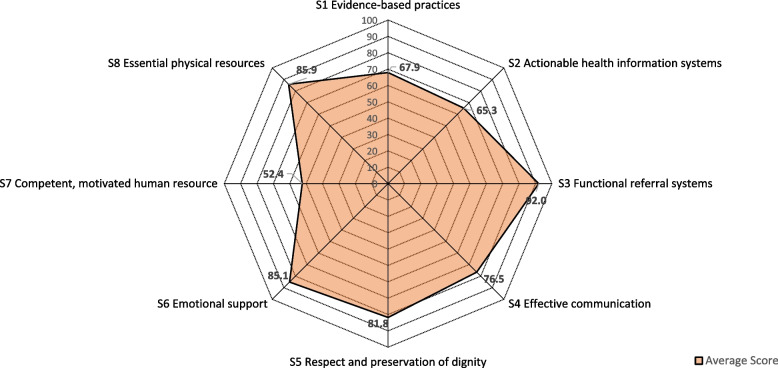
Weighted average score for each of the standards of care of all normal low risk deliveries at health facilities in Nepal

The radar diagram (Fig. 2) shows variation in the weighted average score of all deliveries for the eight standards of care. The bigger the shape of the polygon, the better the standards of care received by the women at HFs. The average score of deliveries was highest (92.0%) for S3 “functional referral system”, and lowest (52.4%) for S7 “competent, motivated human resources”.

### Standards of care, contextual factors, and women’s satisfaction with delivery services (objective 2)

**Fig. 3:**
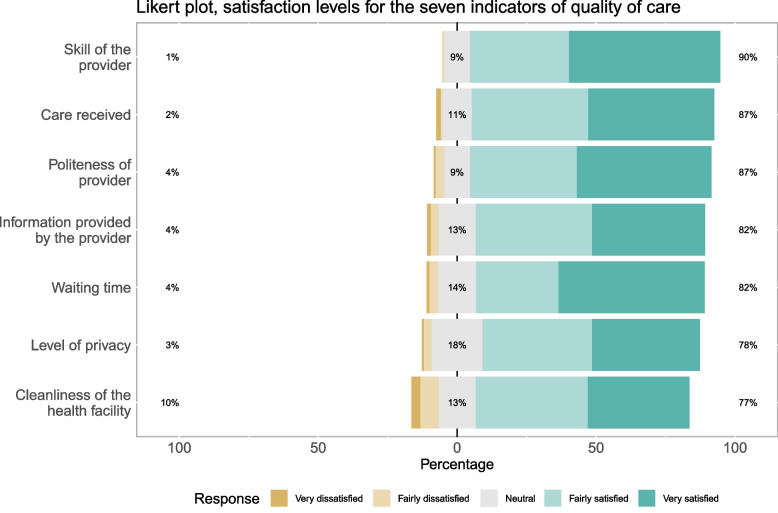
Satisfaction levels of women who had normal low-risk deliveries on the seven indicators of quality of care. Women’s satisfaction was measured on a 5-point Likert Scale, from very dissatisfied to very satisfied

In this study, 46.0% of women were very satisfied, 37.9% fairly satisfied, and 12.2% were neutral with the delivery services they received from the HFs; less than 5 % of the women were very or fairly dissatisfied. With regards to individual aspects of satisfaction, as shown in Fig. 3, a relatively higher proportion of women was very satisfied with waiting time (55.4%), provider’s skill (54.8%), and politeness of the provider (49.3%). Care received, cleanliness, and privacy were the three areas where a relatively higher proportion of respondents, 4 out of 10 women, were fairly satisfied.

**Fig. 4:**
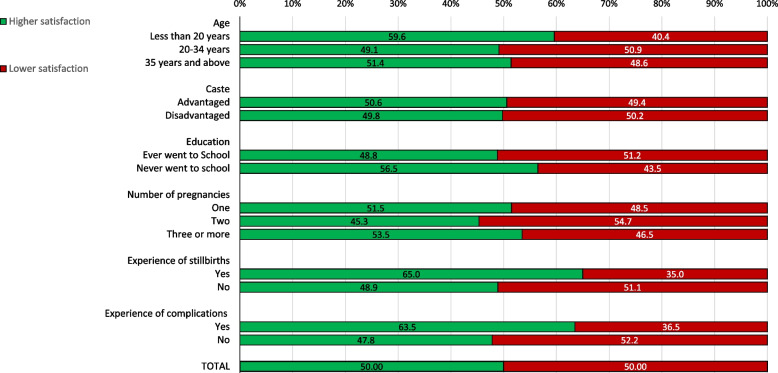
Weighted proportion of women’s satisfaction level with delivery services, in total and by characteristics of women

Figure 4 shows the composite satisfaction level, dichotomized as higher satisfaction vs. lower satisfaction, in total and according to the women’s characteristics. By design, in total 50.0% reported higher satisfaction and 50.0% reported lower satisfaction. Young women, women who had experienced stillbirths in their previous pregnancies, or who had experienced complications during the current pregnancy reported higher levels of satisfaction compared to their counterparts.

The multi-collinearity test did not find a strong correlation among the 65 covariates (53 standards of care and 12 contextual factors) selected for analysis. The bivariate logistic regression analyses carried out for each of the 65 covariates and the outcome variable, reported in Additional file 1: Table S2, found eight variables significantly associated with women’s satisfaction levels. As reported in Table 5, women who delivered at HFs implementing the Maternity Incentive Scheme, having maternity waiting rooms, and having information material had lower odds of being in the higher satisfaction category compared to women who delivered at HFs that did not feature these characteristics. Women who were attended by a service provider when they called for support, who experienced caring behavior from providers and good levels of audio-visual privacy, who delivered in HFs that displayed health statistics and that had delivery care guidelines had higher odds of being in the higher satisfaction category compared to their counterparts. In the multivariate logistic regression model, all but one of these eight variables (i.e., delivery care guidelines) retained their significance. For the Maternity Incentive Scheme, the influence increased in the multivariate model compared to the bivariate model. The influence also increased among HFs displaying health statistics, and for the maternity waiting room, although only minimally.

**Table 5:** (Weighted) bivariate and multivariate logistic regression for assessing standards of care and contextual factors associated with women’s satisfaction with normal low-risk delivery services

Independent variables: deliveries meeting the following criteria at health facilities	Bivariate logistic regression				Multivariate logistic regression			
	OR	95% CI for OR		*p*-value	AOR	95% CI for AOR		*p*-value
		Lower	Upper			Lower	Upper	
**Contextual variables**								
Health facility that implemented the Maternity Incentive Scheme	0.34	0.18	0.65	0.0010**	0.27	0.13	0.55	0.0004*
**Standards of care variables**								
Health facility with any delivery care guidelines	2.07	1.12	3.84	0.0210*	1.97	0.98	3.96	0.0575
Health facility that displayed health statistics	2.15	1.37	3.38	0.0010*	3.16	1.87	5.33	< 0.0001*
Health facility with at least one unit of information materials on maternal care	0.54	0.33	0.86	0.0100*	0.57	0.33	0.97	0.0393*
Health facility with a maternity waiting room	0.36	0.16	0.82	0.0140*	0.35	0.15	0.82	0.0156*
Woman experienced caring and appropriate behavior from the provider	2.67	1.19	5.99	0.0170*	2.59	1.06	6.30	0.0359*
Woman felt comfortable with visual and auditory privacy	2.41	1.28	4.55	0.0070**	2.13	1.04	4.38	0.0399*
Woman was attended to by a provider when she called	8.13	2.04	32.35	0.0030**	5.29	1.18	23.64	0.0292*

*Statistically significant *p* < 0.05

*OR* odds ratio, *AOR* adjusted odds ratio, *CI* confidence interval

## Discussion

### Key findings and locating them in the literature

#### Health facilities’ compliance with standards of care

Compliance with the eight standards of care for normal low-risk deliveries in HFs in Nepal varied and was inconsistent across the 53 indicators analyzed. A few indicators, such as the provider completing the discharge slip, the provider administering uterotonics, and the provider cleaning and drying the newborn, were met for all or almost all deliveries. Several other indicators were met for a considerable proportion of the deliveries, but some indicators were only met for a small proportion of deliveries. The output indicators of the standards of care performed marginally better than the input indicators, reflecting the wide gaps in the structural components of care, which block pathways to meeting the standards of both the process of care and the experience of care. Some of the input indicators, such as the availability of client feedback systems and delivery care guidelines at HFs and the availability of job descriptions with providers, scored very low, which lowered the overall performance of the input indicators. Of the eight standards of care, S1 “evidence-based practice” showed mixed results. While essential equipment and medicines for mothers and equipment for newborns were frequently available for most deliveries, the delivery care guidelines, trained providers, and essential medicines for newborns were unavailable for many deliveries. Gaps in structural factors of QoC are common across LMICs; for example, in Ethiopia, one-third of HFs assessed in 2022 had low readiness to provide routine labor and delivery care, with only 52.2% of the hospitals having essential medicines, equipment, and supplies [16]. While the average score for S2 “actionable health information systems” was 65.3%, S3 “functional referral system” was almost universally achieved. For example, a functional ambulance service was available for 94.5% of deliveries. This contrasts with much lower scores in Madagascar in 2016, where this ranged from 3.1% for basic health centers to 83.3% for university (referral) hospitals [17], and in Nigeria, where none of the 60 primary health care centers assessed in 2020 had a functional ambulance [18]. The higher availability of functional equipment for referral in Nepal, however, does not guarantee a functional referral system as shown in a 2021 study reporting that 33.2% of maternal deaths in Nepal are attributable to delays in reaching the HF [7].

S4 “effective communication” performed moderately well; for example, the availability of a 24-hour on-call service was met for nine out of 10 deliveries in Nepal, much higher than in Tanzania, where only 28.3% of HFs met this standard in 2019 [19]. Most deliveries met the indicators of S5 “respect and preservation of dignity”, indicating that women giving birth in HFs in Nepal generally experience kind, considerate, and appropriate behaviors from providers. However, most HFs lacked a system to gather client feedback. In this study, nine out of 10 women did not experience any discriminatory behavior from their providers, which supports the findings from another recent nationally representative survey reporting that during their most recent birth only 3.8 and 13.8% of women experienced physical and verbal abuse, respectively [4]. Provider misbehavior in Nepal is much lower compared to India, where a systematic review published in 2020 found that 25.7, 16.9, and 14.7% of women experienced verbal abuse, physical abuse, and discrimination, respectively [20]. Similarly, in Ethiopia 36.0% of women experienced mistreatment by the provider during childbirth, according to a cross-sectional study published in 2017 [21].

With regards to S6 “emotional support”, most providers were empathetic, 94.0% allowed women to have their companion present when requested, and 71.0% provided them with emotional support and reassurance. Compliance with emotional support is higher in Nepal compared to other LMICs; for example, a 2019 study from Uttar Pradesh, India, showed that nearly a quarter of women interviewed were not allowed to have a companion close to them during labour and delivery [22]. Compliance with the five indicators of S7 “competent, motivated human resources” varied, with most providers attending the deliveries not having a clear job description, but nine out of 10 HFs having routine quality assurance activities. Similarly, most deliveries met eight out of nine indicators of S8 “essential physical resources”. The indicator on IPC was met for only six out of 10 deliveries. Studies from LMICs often show important gaps in physical resources, such as toilets, for clients [23].

#### Standards of care as determinants of women’s satisfaction with delivery services

Six of the 53 standards of care indicators analyzed in this study were found to be statistically significant in influencing women’s satisfaction with delivery services. Standards of care related to the inter-personal communication of providers with clients—the caring behavior—were associated with higher satisfaction levels among women. This finding aligns with the findings of a cross-sectional study carried out in Iraq in 2019, where the provider’s good behavior during delivery was related to women’s satisfaction with the services [24]. Women’s experiences of privacy during labour and delivery were also related to higher satisfaction levels in Nepal. This is consistent with studies carried out in other LMICs. A systematic review from 2015 shows that women treated with dignity, respect, and courtesy were more satisfied [25], while another systematic review focused on Ethiopia reports that two-thirds of Ethiopian women were satisfied with skilled delivery care, which was correlated with privacy and short waiting times [26]. Studies show notable differences in the factors influencing satisfaction among Asian and African women, where Asian women preferred providers’ behavior over their technical competence, but African women preferred providers’ confidence and competence over their behavior [25]. In view of the high reported compliance with dignified and respectful delivery care for women, the findings of this study suggest that providers in Nepal are more empathetic compared to many other LMICs. This could be due to cultural factors, with Nepalese women tending to highly value health providers and therefore responding positively. Similarly, Nepalese women may not be aware of the standards of care and have relatively low expectations.

The display of health statistics at the HF, where women delivered, emerged as a strong predictor of higher satisfaction; surprisingly, the availability of information materials on maternal care at the HF emerged as a predictor of lower satisfaction. Most of the women in this study were relatively educated, and it may be that the health statistics displayed at HFs attracted the women who could read them, thereby contributing to higher satisfaction levels. In Nepal, displaying health statistics is an approach pursued for promoting evidence-based management of health programs. Although the information materials were available in the HF, women’s access to them could have been low, or the materials were not attractive. Another explanation could be that displaying health statistics might have been perceived as more modern or a means of quality control and assurance, and the availability of information materials might have been perceived as more old-fashioned.

Similarly, nine out of 10 deliveries in this study took place in HFs that had maternity waiting rooms, and most of the women benefiting from these had lower satisfaction levels than women in HFs without maternity waiting rooms. Although this study did not analyze the comfort and quality of the maternity waiting rooms, it is possible that just having a room is not adequate. Comfort and physical facilities are important for satisfaction; for example, a recent Ethiopian study found that providing secure and comfortable waiting rooms can reduce the desire for home delivery and improve client satisfaction [27]. An alternative explanation may be that HFs that have a waiting room tend to be larger and often overcrowded and may thus keep women in the waiting room for prolonged periods of time, with little or no attention and support from providers.

#### Contextual factors as determinants of women’s satisfaction with delivery services

Of the 12 contextual factors analyzed, only the Maternity Incentive Scheme showed a statistically significant association with women’s satisfaction with delivery services. Most deliveries in this study took place in public hospitals that implemented the Maternity Incentive Scheme, which was associated with lower satisfaction among women. Although this scheme has been key to addressing the financial barriers to accessing HFs across Nepal, operational challenges such as appropriate monitoring of women’s antenatal visits, delays in providing the transportation incentives to women and stockouts of essential medicines and supplies at HFs [28] are likely to decrease levels of satisfaction. A study published in 2018 found that only 43% of women delivering in public hospitals in Nepal were very satisfied with the transportation incentives [29], while a study published in 2021 showed that less than two-thirds of women delivering at a HF knew about the scheme [6].

### Policy, program and research implications

Compliance with the standards of care at HFs and several other factors determine women’s satisfaction levels, and these in turn affect how often they and their family members, friends and other acquaintances use HFs to give birth. In Nepal, a people-centered approach to service provision that monitors the process of care, and the experience of care has been emphasized. Using client satisfaction as a measure of QoC is necessary but often incomplete and contentious as clients in LMICs often have low expectations of the healthcare they receive [30]. Relying solely on client satisfaction as a measure of the QoC may yield high levels of satisfaction despite HFs not meeting the standards of care and objectives for quality improvement. Hence, to understand QoC holistically, Nepal must complement the indicators of client satisfaction with other indicators that gauge the level of adherence to the standards of care, and such data needs to be collected and reported more frequently. For example, in addition to the MSS, Nepal needs to contextualize and use the globally available quality improvement tools that monitor the knowledge and skills of providers. A pool of mentors and coaches who can visit the HFs, assess provider knowledge and skills, and provide feedback, needs to be developed at the local level. Compliance with standards of care varied for different indicators in Nepal, but the results are encouraging. With the improving policy environment, QoC has received greater attention in the newly endorsed Nepal Health Sector Strategic Plan (NHS-SP) 2023–2030. With the implementation of the various quality improvement initiatives, such as the MSS and Maternal and Perinatal Death Surveillance and Response, progress in compliance with standards of care and improvements in QoC can be expected. The WHO’s standards of care for maternal and newborn care during childbirth are exceedingly comprehensive; therefore, full compliance with these standards is a challenge for LMICs, and Nepal is no exception. Nepal needs to contextualize these standards of care and integrate its monitoring into the routine information management systems. In the new federalized context, resources are available at all three tiers of government, which must be rationally allocated and well-coordinated for developing human resource capacity to implement quality improvement initiatives, monitor compliance with the standards of care and use data to feed into the planning and quality improvement cycle.

In this study, nine out of 10 births occurred at hospitals, which indicates low utilization of the other peripheral public HFs that offer normal, low-risk delivery services. Several of the birthing HFs in Nepal are not strategically located and, therefore, do not attract clients, resulting in clients bypassing the local HFs and using big referral hospitals for normal, low-risk delivery services [31]. This tendency has an enormous impact on the functionality of the HFs and on their adherence to the standards of care. Bigger hospitals are often overwhelmed due to client overcrowding, as well as medicines and supplies often being stocked out, and the providers cannot take good care of the clients, resulting in low or no client satisfaction. Moving forward, it is critically important to rationalize the establishment of new HFs and ensure appropriate quality of care of the existing HFs to build trust and attract more women to use HFs for delivery services. Client and community education is equally important to increase the uptake of normal low-risk delivery services at peripheral public HFs. In the new federal system of government, the local governments have an important role in improving the management of normal low-risk delivery services. The Nepal government is committed to providing free maternal health care services to women. In this purview, it is important to review the operational challenges of the Maternity Incentive Scheme to create demand for HF-based maternal health services, gain trust from the community, and improve service quality and women’s satisfaction.

### Strengths and limitations of the study

This study is the first of its kind conducted in Nepal and provides multiple perspectives on the standards of care for normal, low-risk deliveries using the WHO framework for the quality of maternal and newborn health care. This study has several strengths and some limitations.

#### Study design

This study used a conceptual framework-led approach informed by the WHO framework and assessed standards of care from multiple perspectives. It used data from the recent nationally representative NHFS that included all public HF types and private hospitals, comprising inventory assessments, provider interviews, observations of labor and delivery and client exit interviews. The NHFS, was, however not specifically designed for the research question addressed in this study. As a result, several variables of interest were missing. Moreover, while the HFs included in this study were nationally representative, observations of women giving birth were not. In fact, relying on only 2 days of observations of women’s labour and delivery per HF may have created sampling bias, potentially leading to more large facilities represented in our sample.

#### Data collection

Validated, standardized, and pre-tested tools were used in accordance with the quality standards of the NHFS [6]. Data were collected by trained nurses, medical officers, and public health professionals; a computer-based data collection system employing various data checks ensured high quality data. Limitations include the low number of cases observed at lower-level HFs and private hospitals, which limited informative analyses for different types of HFs. Women were observed at different stages of labor; therefore, complete information on labour and delivery was not available for all women analyzed.

#### Data analysis

Study variables were carefully selected from the large data set following a largely a priori approach in line with the conceptual framework of the study. Various composite measures were developed to reduce the large number of variables, allowing for more meaningful analyses and avoiding power limitations; collinearity checks were carried out. The study focused on maternal care-specific indicators and did not focus on newborn-specific indicators, as recommended by the WHO.

## Conclusions

The learnings from the first half of the SDG-relevant period imply that Nepal needs to urgently accelerate and sustain the quality improvement efforts to meet target 3.1 of reducing MMR to 70 deaths per 100,000 live births. Improving coverage of maternal health services and meeting standards of care are the prerequisites for achieving this target. Although Nepal is performing moderately well with regards to meeting the standards of normal low-risk delivery care, gaps exist in critical components such as human resource capacity, strengthened supply chain systems, a supportive environment for provider motivation, and quality improvement systems to collect and address client feedback. These gaps are challenging the implementation of the Maternity Incentive Scheme, a key intervention for Nepal to improve maternal survival. Moving forward, all three tiers of government—federal, provincial, and local—should strengthen collaboration, tap into and mobilize available resources, and implement high-impact integrated programs. Measuring compliance with standards of care should be integral to routine information management systems to assess and track progress.

### Supplementary Information

**Additional file 1: Table S1.** Weighted number and proportion of deliveries (including 95% CI) meeting the input, output, and all indicators by the standards of care, and the average score by the standards of care among all women observed and interviewed (*n* = 320). **Table S2.** (Weighted) bivariate logistic regression for assessing the factors associated with women’s satisfaction with normal low-risk delivery services (with Odds Ratio (OR) and 95% CI for OR).

**Additional file 2: Fig. S1.** Weighted proportion of deliveries meeting the 53 indicators among all women observed and interviewed arranged in descending order (*n* = 320).

## Data Availability

The data used in the study is publicly available at the DHS Program website: https://dhsprogram.com/Data/

## Abbreviations

AOR: Adjusted odds ratio
BEmONC: Basic emergency obstetric and neonatal care
CEmONC: Comprehensive emergency obstetric and neonatal care
CI: Confidence interval
COVID: Coronavirus disease
EmONC: Emergency obstetric and neonatal care
HF: Health facility
IBM: International business machines corporation
IPC: Infection prevention and control
LMU: Ludwig-maximilians-universität
LMIC: Low and middle-income countries
MDG: Millennium development goals
MMR: Maternal mortality ratio
MSS: Minimum service standards
NHS-SP: Nepal health sector - strategic plan
NHFS: Nepal health facility survey
NHRC: Nepal health research council
OR: Odds ratio
PCA: Principal component analysis
QoC: Quality of care
S: Standard
SDG: Sustainable development goal
SPSS: Statistical package for the social sciences
UN: United nations
VIF: Variation inflation factor
WHO: World health organization
