# Supplementary material for: Standards of care and determinants of women’s satisfaction with delivery services in Nepal: a multi-perspective analysis using data from a health facility-based survey
Source: BMC Pregnancy Childbirth. 2024 Feb 13;24:132. doi: 10.1186/s12884-024-06301-9 (PMC10863287; doi:10.1186/s12884-024-06301-9)
Supplement: Supplementary file 1 — Additional file 1: Table S1. Weighted number and proportion of deliveries (including 95% CI) meeting the input, output, and all indicators by the standards of care, and the average score by the standards of care among all women observed and interviewed (n = 320). Table S2. (Weighted) bivariate logistic regression for assessing the factors associated with women’s satisfaction with normal low-risk delivery services (with Odds Ratio (OR) and 95% CI for OR). [file 12884_2024_6301_MOESM1_ESM.docx]

**Additional File 1**

**Table S1: Weighted number and proportion of deliveries (including 95% CI) meeting the input, output, and all indicators by the standards of care, and the average score by the standards of care among all women observed and interviewed (n=320)**

| **Standards** | | **Number (320)** | **Percent** | **95% CI** |
| --- | --- | --- | --- | --- |
| **Standard 1. Evidence-based practices** | | | | |
| Input | Health facility with at least one functioning unit of each of seven basic equipment and supplies for mothers | 302 | 94.5 | [92.0; 97.0] |
|  | Health facility with any delivery care guidelines | 52 | 16.3 | [12.2; 20.3] |
|  | Health facility with at least one provider trained on delivery care | 192 | 59.9 | [54.5; 65.3] |
|  | Health facility with five essential medicines for mothers | 289 | 90.2 | [87.0; 93.5] |
|  | Health facility with two types of equipment for an assisted delivery | 243 | 75.9 | [71.2; 80.6] |
|  | Health facility with three basic types of equipment for newborns | 299 | 93.6 | [90.9; 96.3] |
|  | Health facility with five essential medicines for newborns | 35 | 10.8 | [7.0; 14.0] |
|  | ***Average score of all seven input indicators*** | ***-*** | ***63.0*** | ***[62.2, 64.9]*** |
| Output | Provider monitored the mother’s vital signs | 171 | 53.5 | [48.0; 59.0] |
|  | Provider administered immediate postpartum uterotonic | 318 | 99.3 | [98.4; 100.0] |
|  | Provider dried, covered, and cleaned the newborn | 319 | 99.6 | [98.9; 100.0] |
|  | Provider delivered the newborn to the mother’s abdomen | 248 | 77.5 | [72.9; 82.1] |
|  | Provider supported the initiation of early breastfeeding | 168 | 52.6 | [47.1; 58.1] |
|  | Provider checked newborn breathing and crying | 305 | 95.4 | [93.1; 97.7] |
|  | Provider helped mother to initiate skin-to-skin contact | 242 | 75.6 | [70.9; 80.3] |
|  | Provider counseled on postpartum family planning | 76 | 23.6 | [19.0; 28.3] |
|  | ***Average score of all eight output indicators*** | ***-*** | ***72.1*** | ***[70.5, 73.8]*** |
|  | ***Average score of all indicators of Standard 1*** | ***-*** | ***67.6*** | ***[66.3, 68.9]*** |
| **Standard 2. Actionable health information systems** | | | | |
| Input | Health facility with Maternal and Newborn Health (MNH) service register | 212 | 66.4 | [61.2; 71.6] |
|  | Health facility with availability of HMIS monthly reports | 207 | 64.6 | [59.4; 69.9] |
|  | Health facility that displayed health statistics | 136 | 42.4 | [37.0; 47.9] |
|  | Health facility with Quality Assurance Action Plans | 169 | 52.9 | [47.4; 58.4] |
|  | ***Average score of all four input indicators*** | ***-*** | ***56.6*** | ***[53.8, 59.4]*** |
| Output | Woman who had a completed discharge slip | 320 | 100 | - |
|  | ***Average score of all indicators of Standard 2*** | ***-*** | ***65.3*** | ***[63.0, 67.5]*** |
| **Standard 3. Functional referral systems** | | | | |
| Input | Health facility with at least one unit of functioning ambulance or emergency transport | 303 | 94.5 | [92.0; 97.0] |
|  | Health facility with at least one type of communication equipment | 287 | 89.7 | [86.3; 93.0] |
|  | ***Average score of the two input indicators*** | ***-*** | ***92.1*** | ***[90.0, 94.2]*** |
| Output | None | - | - | - |
|  | ***Average score of all indicators of Standard 3*** | ***-*** | ***92.1*** | ***[90.0, 94.2]*** |
| **Standard 4. Effective communication** | | | | |
| Input | Health facility with at least one unit of information materials on maternal care | 216 | 67.6 | [62.5; 72.8] |
|  | Health facility that received at least one external supervision from federal, provincial, or local authorities in the last 12-month-period before data collection | 310 | 97.0 | [95.1; 98.9] |
|  | Health facility which had a call list for 24-hour service | 294 | 92.0 | [89.0; 95.0] |
|  | ***Average score of all three input indicators*** | ***-*** | ***85.5*** | ***[83.5, 87.6]*** |
| Output | Woman received postnatal counseling before discharge | 235 | 73.4 | [68.5; 78.3] |
|  | Woman received information about the delivery procedures | 224 | 70.1 | [65.1; 75.2] |
|  | Provider completed a partograph | 187 | 58.6 | [53.1; 64.0] |
|  | ***Average score of all three output indicators*** | ***-*** | ***67.4*** | ***[64.4, 70.4]*** |
|  | ***Average score of all indicators of Standard 4*** | ***-*** | ***76.5*** | ***[74.6, 78.3]*** |
| **Standard 5. Respect and preservation of dignity** | | | | |
| Input | Health facility with physical environment that allows privacy | 306 | 95.5 | [93.3; 97.8] |
|  | Health facility with a system for collecting clients' opinion | 53 | 16.7 | [12.6; 20.8] |
|  | ***Average score of the two input indicators*** | ***-*** | ***56.1*** | ***[53.7, 58.6]*** |
| Output | Woman did not experience use of physical force or abrasive behavior from the provider | 316 | 98.9 | [98.0; 100.0] |
|  | Woman experienced caring and appropriate behavior from the provider | 289 | 90.3 | [87.0; 93.5] |
|  | Woman felt comfortable with visual and auditory privacy | 269 | 84.1 | [80.1; 88.2] |
|  | Woman did not experience any discriminatory behavior from the provider | 306 | 95.7 | [93.4; 98.0] |
|  | Woman was attended to by a provider when she called | 300 | 93.9 | [91.2; 97.0] |
|  | Woman was not scolded by any provider | 254 | 79.2 | [75.0; 84.0] |
|  | ***Average score of all six output indicators*** | ***-*** | ***90.4*** | ***[88.9, 91.8]*** |
|  | **Average score of all indicators of Standard 5** | ***-*** | ***81.8*** | ***[80.5, 83.0]*** |
| **Standard 6. Emotional support** | | | | |
| Input | Health facility with a maternity waiting room | 289 | 90.3 | [87.0; 93.5] |
| Output | Woman who was allowed a companion to join her when requested | 301 | 94.0 | [91.4; 96.6] |
|  | Provider who provided emotional support and reassurance to the woman | 227 | 71.0 | [66.0; 76.0] |
|  | ***Average score of both output indicators*** | ***-*** | ***82.5*** | [79.8, 85.2] |
|  | **Average score of all indicators of Standard 6** | ***-*** | ***85.1*** | ***[83.1, 87.1]*** |
| **Standard 7. Competent, motivated human resources** | | | | |
| Input | Provider received supervision in the last 12 months | 204 | 63.8 | [58.5; 69.1] |
|  | Health facility that implemented quality assurance activities routinely | 290 | 90.5 | [87.3; 93.8] |
|  | Provider reported having a written job description | 14 | 4.5 | [2.2; 6.7] |
|  | Provider reported opportunities for staff promotion | 78 | 24.5 | [19.7; 29.2] |
|  | ***Average score of all four input indicators*** | ***-*** | ***45.8*** | ***[43.8, 47.9]*** |
| Output | Provider who was experienced | 252 | 78.7 | [74.2; 83.2] |
|  | ***Average score of all indicators of Standard 7*** | ***-*** | ***52.4*** | ***[50.5, 54.3]*** |
| **Standard 8. Essential physical resources** | | | | |
| Input | Health facility with a regular source of electricity | 311 | 97.3 | [95.6; 99.1] |
|  | Health facility with basic water supply in maternity care areas | 311 | 97.2 | [95.4; 99.1] |
|  | Health facility with one functioning unit of the six-infection prevention and control equipment | 191 | 59.6 | [54.1; 65.0] |
|  | Health facility with a healthcare waste management system | 269 | 83.9 | [79.9; 88.0] |
|  | Health facility with a toilet for female clients | 255 | 79.7 | [75.3; 84.1] |
|  | Health facility with a newborn corner | 254 | 79.3 | [74.8; 83.7] |
|  | ***Average score of all six input indicators*** | ***-*** | ***82.8*** | ***[80.8, 84.9]*** |
| Output | Woman who reported access to drinking water | 256 | 80.1 | [76.0; 84.5] |
|  | Woman who reported access to a toilet | 313 | 97.7 | [96.1; 99.4] |
|  | Woman who reported getting maternity bed on time | 315 | 98.6 | [97.3; 99.9] |
|  | ***Average score of all three output indicators*** | ***-*** | ***92.1*** | ***[90.4, 93.9]*** |
|  | ***Average score of all indicators of Standard 8*** | ***-*** | ***85.9*** | ***[84.4, 87.5]*** |

Note: These indicators were selected from the 2021 Nepal Health Facility Survey data set. The selection of indicators was guided by WHO’s “Standards for Improving the Quality of Maternal and Newborn Care in Health Facilities 2016.”

| **Additional File 1**  **Table S2: (Weighted) bivariate logistic regression for assessing the factors associated with women’s satisfaction with normal low-risk delivery services (with Odds Ratio (OR) and 95% CI for OR)** | | | | |
| --- | --- | --- | --- | --- |
| **Co-variates** | **OR** | **95% CI for OR** | | **p-value** |
|  |  | **Lower** | **Upper** |  |
| **Women’s characteristics** |  | | | |
| Age (Ref 35 or more years) |  |  |  |  |
| Less than 20 years | 0.719 | 0.188 | 2.756 | 0.6303 |
| 20-34 years | 1.097 | 0.363 | 3.316 | 0.8691 |
| Caste: Advantaged (Ref Disadvantaged) | 1.033 | 0.620 | 1.720 | 0.9016 |
| Education: Ever attended school (Ref = No) | 0.734 | 0.401 | 1.343 | 0.3154 |
| Number of pregnancies (Ref Three or more) |  |  |  |  |
| One | 1.081 | 0.621 | 1.880 | 0.7840 |
| Two | 1.386 | 0.766 | 2.508 | 0.2806 |
| Experience of complications | 1.898 | 0.994 | 3.624 | 0.0522 |
| Experience of stillbirths | 1.940 | 0.796 | 4.729 | 0.1448 |
|  |  |  |  |  |
| **Service provider characteristics** |  | | | |
| Type of provider (Ref Auxiliary Nurse Midwives) |  |  |  |  |
| Doctor | 0.419 | 0.156 | 1.127 | 0.0849 |
| Nurse | 1.144 | 0.719 | 1.820 | 0.5698 |
| Sex of provider assisting birth (female) (Ref Male) | 0.559 | 0.151 | 2.075 | 0.3846 |
|  |  |  |  |  |
| **Health facility characteristics** |  | | | |
| Health facility type (Ref Private hospitals) |  |  |  |  |
| Public hospital | 1.611 | 0.932 | 2.784 | 0.0877 |
| Other public health facility | 1.045 | 0.404 | 2.701 | 0.9280 |
| Health facility that implemented the Maternity Incentive Scheme (Ref No) | 0.336 | 0.175 | 0.645 | 0.0011* |
| Type of site (Ref EmONC sites) |  |  |  |  |
| Comprehensive EmONC sites | 1.616 | 0.534 | 4.886 | 0.3952 |
| Basic EmONC sites | 0.410 | 0.054 | 3.126 | 0.3894 |
| Distance to HF- proximal (Ref Semi-proximal) | 1.414 | 0.893 | 2.239 | 0.1399 |
|  |  |  |  |  |
| **Standard 1: Evidence-based practices** |  | | | |
| ***Input indicators*** |  |  |  |  |
| Health facility with at least one functioning unit of each of seven basic equipment and supplies for mothers (Ref No) | 0.617 | 0.230 | 1.651 | 0.3359 |
| Health facility with any delivery care guidelines (Ref No) | 2.069 | 1.116 | 3.838 | 0.0210* |
| Health facility with five essential medicines for mothers (Ref No) | 0.730 | 0.347 | 1.538 | 0.4079 |
| Health facility with two types of equipment for an assisted delivery (Ref No) | 1.089 | 0.652 | 1.818 | 0.7438 |
| Health facility with three basic types of equipment for newborns (Ref No) | 0.578 | 0.230 | 1.452 | 0.2433 |
| Health facility with five essential medicines for newborns (Ref No) | 0.704 | 0.345 | 1.435 | 0.3334 |
| ***Output indicators*** |  |  |  |  |
| Provider monitored the mother’s vital signs (Ref No) | 0.713 | 0.459 | 1.108 | 0.1323 |
| Provider administered immediate postpartum uterotonic (Ref No) | 0.383 | 0.020 | 7.355 | 0.5246 |
| Provider dried, covered, and cleaned the newborn (Ref No) | 0.678 | 0.020 | 22.686 | 0.8283 |
| Provider delivered the newborn to the mother’s abdomen (Ref No) | 1.078 | 0.638 | 1.823 | 0.7787 |
| Provider supported the initiation of early breastfeeding (Ref No) | 1.445 | 0.930 | 2.246 | 0.1015 |
| Provider checked newborn breathing and crying (Ref No) | 2.033 | 0.676 | 6.120 | 0.2068 |
| Provider helped mother to initiate skin-to-skin contact (Ref No) | 1.110 | 0.666 | 1.849 | 0.6896 |
| Provider counseled on postpartum family planning (Ref No) | 0.965 | 0.576 | 1.616 | 0.8924 |
|  |  |  |  |  |
| **Standard 2: Actionable health information systems** |  | | | |
| ***Input indicators*** |  |  |  |  |
| Health facility with Maternal and Newborn Health Service Register (Ref No) | 0.668 | 0.419 | 1.065 | 0.0902 |
| Health facility with Health Management Information System monthly reports (Ref No) | 0.753 | 0.475 | 1.193 | 0.2264 |
| Health facility that displayed health statistics (Ref No) | 2.147 | 1.366 | 3.375 | 0.0009* |
| Health facility with Quality Assurance Action Plans (Ref No) | 0.866 | 0.558 | 1.344 | 0.5224 |
| ***Output indicators*** |  |  |  |  |
| Woman with a completed discharge slip | - | - | - |  |
|  |  |  |  |  |
| **Standard 3: Functional referral systems** | | | | |
| **Input indicators** |  |  |  |  |
| Health facility with at least one unit of functioning ambulance or emergency transport (Ref No) | 0.877 | 0.334 | 2.303 | 0.7895 |
| Health facility with at least one type of communication equipment (Ref No) | 1.104 | 0.537 | 2.270 | 0.7870 |
|  |  |  |  |  |
| **Standard 4: Effective communication** | | | | |
| **Input indicators** |  |  |  |  |
| Health facility with at least one unit of information materials on maternal care (Ref No) | 0.535 | 0.332 | 0.861 | 0.0101* |
| Health facility that received at least one external supervision from federal, provincial, or local authorities in the last 12-month-period before data collection (Ref No) | 0.320 | 0.073 | 1.405 | 0.1311 |
| Health facility which had a availability of a 24-hour on-call service (Ref No) | 0.690 | 0.304 | 1.564 | 0.3739 |
| **Output indicators** |  |  |  |  |
| Women received postnatal counseling before discharge (Ref No) | 1.566 | 0.949 | 2.585 | 0.0794 |
| Provider explained to women about the delivery procedures (Ref No) | 1.459 | 0.901 | 2.363 | 0.1241 |
| Provider completed a partograph (Ref No) | 0.692 | 0.443 | 1.082 | 0.1067 |
|  |  |  |  |  |
| **Standard 5: Respect and preservation of dignity** | | | | |
| **Input indicators** |  |  |  |  |
| Health facility with physical environment that allows privacy (Ref No) | 0.839 | 0.289 | 2.432 | 0.7460 |
| Health facility with a system for collecting clients' opinions (Ref No) | 1.407 | 0.778 | 2.545 | 0.2585 |
| **Output indicators** |  |  |  |  |
| Woman did not experience use of physical force or abrasive behavior from the provider (Ref No) | 3.579 | 0.294 | 43.534 | 0.3172 |
| Woman experienced caring and appropriate behavior from the provider (Ref No) | 2.674 | 1.194 | 5.991 | 0.0168* |
| Woman felt comfortable with visual and auditory privacy (Ref No) | 2.412 | 1.278 | 4.553 | 0.0066* |
| Woman did not experience discriminatory behavior from the provider (Ref No) | 1.226 | 0.416 | 3.612 | 0.7118 |
| Woman was attended to by a provider when she called (Ref No) | 8.130 | 2.043 | 32.350 | 0.0029* |
| Women was not scolded by any provider (Ref No) | 1.593 | 0.921 | 2.755 | 0.0955 |
|  |  |  |  |  |
| **Standard 6: Emotional support** | | | | |
| **Input indicators** |  |  |  |  |
| Health facility with availability of maternity waiting room (Ref No) | 0.363 | 0.161 | 0.817 | 0.0143* |
| **Output indicators** |  |  |  |  |
| Woman who was allowed a companion to join her when requested (Ref No) | 1.816 | 0.696 | 4.737 | 0.2225 |
| Provider who provided emotional support and reassurance to the woman (Ref No) | 1.010 | 0.623 | 1.637 | 0.9672 |
|  |  |  |  |  |
| **Standard 7: Competent, motivated human resources** | | | | |
| **Input indicators** |  |  |  |  |
| Provider received supervision in the last 12 months (Ref No) | 1.342 | 0.849 | 2.120 | 0.2076 |
| Health facility that implemented quality assurance activities routinely (Ref No) | 0.739 | 0.347 | 1.571 | 0.4316 |
| Provider reported having a written job description (Ref No) | 2.088 | 0.678 | 6.430 | 0.1997 |
| Provider reported opportunities for staff promotion (Ref No) | 1.265 | 0.758 | 2.109 | 0.3683 |
| **Output indicators** |  |  |  |  |
| Provider who was experienced (Ref No) | 0.958 | 0.561 | 1.637 | 0.8756 |
|  |  |  |  |  |
| **Standard 8: Essential physical resources** | | | | |
| **Input indicators** |  |  |  |  |
| Health facility with a regular source of electricity (Ref No) | 0.277 | 0.054 | 1.414 | 0.1226 |
| Health facility with basic water supply in maternity care areas (Ref No) | 0.125 | 0.016 | 1.000 | 0.0500 |
| Health facility with one functioning unit of the six-infection prevention and control equipment (Ref No) | 1.364 | 0.872 | 2.135 | 0.1741 |
| Health facility with health-care waste management system (Ref No) | 1.266 | 0.696 | 2.306 | 0.4397 |
| Health facility with a toilet for female clients (Ref No) | 1.063 | 0.616 | 1.833 | 0.8261 |
| Health facility with a newborn corner (Ref No) | 0.795 | 0.462 | 1.367 | 0.4068 |
| **Output indicators** |  |  |  |  |
| Women who reported access to drinking water (Ref No) | 1.654 | 0.947 | 2.889 | 0.0769 |
| Women who reported access to a toilet (Ref No) | 0.675 | 0.152 | 2.990 | 0.6044 |
| Women who reported getting a maternity bed on time (Ref No) | 1.856 | 0.267 | 12.903 | 0.5317 |
|  |  |  |  |  |

*Statistically significant p<0.05.
