# Supplementary material for: Standards of care and determinants of women’s satisfaction with delivery services in Nepal: a multi-perspective analysis using data from a health facility-based survey
Source: BMC Pregnancy Childbirth. 2024 Feb 13;24:132. doi: 10.1186/s12884-024-06301-9 (PMC10863287; doi:10.1186/s12884-024-06301-9)
Supplement: Supplementary file 2 — Additional file 2: Fig. S1. Weighted proportion of deliveries meeting the 53 indicators among all women observed and interviewed arranged in descending order (n = 320). [file 12884_2024_6301_MOESM2_ESM.docx]

**Additional File 2**

**Figure S1: Weighted proportion of deliveries meeting the 53 indicators among all women observed and interviewed arranged in descending order (n=320)**
